# Analysis of the Dose-Response Relationship Between the International Normalized Ratio and Hepatic Encephalopathy in Patients With Liver Cirrhosis Using Restricted Cubic Spline Functions

**DOI:** 10.3389/fpubh.2022.919549

**Published:** 2022-06-28

**Authors:** Juntao Tan, Yuxin He, Zhanbiao Li, Qinghua Zhang, Yanzhi Yang, Qian Xu, Xiaomei Xu

**Affiliations:** ^1^Operation Management Office, Affiliated Banan Hospital of Chongqing Medical University, Chongqing, China; ^2^Department of Medical Administration, Affiliated Banan Hospital of Chongqing Medical University, Chongqing, China; ^3^Department of Science and Education, Affiliated Banan Hospital of Chongqing Medical University, Chongqing, China; ^4^Department of Endocrinology and Metabolism, Chengdu First People's Hospital, Chengdu, China; ^5^College of Medical Informatics, Chongqing Medical University, Chongqing, China; ^6^Medical Data Science Academy, Chongqing Medical University, Chongqing, China; ^7^Library, Chongqing Medical University, Chongqing, China; ^8^Department of Infectious Diseases, The Fifth People's Hospital of Chengdu, Chengdu, China; ^9^Department of Infectious Diseases, The First Affiliated Hospital of Chongqing Medical University, Chongqing, China

**Keywords:** international normalized ratio, liver cirrhosis, hepatic encephalopathy, restricted cubic spline, dose-response

## Abstract

**Background:**

The International Normalized Ratio (INR) is significantly associated with Hepatic Encephalopathy (HE) in patients with liver cirrhosis. However, the dose-response relationship between continuous INR changes and HE risk has not been clearly defined. Thus, our goal was to explore the continuous relationship between HE and INR among patients hospitalized with liver cirrhosis and to evaluate the role of the INR as a risk factor for HE in these patients.

**Methods:**

A total of 6,266 people were extracted from the Big Data Platform of the Medical Data Research Institute of Chongqing Medical University. In this study, unconditional logistic regression and restricted cubic spline (RCS) model were used to analyze the dose-response association of INR with HE. Alcoholic liver disease, smoking status, and drinking status were classified for subgroup analysis.

**Results:**

The prevalence of HE in the study population was 8.36%. The median INR was 1.4. After adjusting for alcoholic liver disease, age, smoking status, drinking status, total bilirubin, neutrophil percentage, total hemoglobin, aspartate aminotransferase, serum sodium, albumin, lymphocyte percentage, serum creatinine, red blood cell, and white blood cell, multivariate logistic regression analysis revealed that INR ≥ 1.5 (OR = 2.606, 95% CI: 2.072–3.278) was significantly related to HE risk. The RCS model showed a non-linear relationship between the INR and HE (non-linear test, χ^2^ = 30.940, *P* < 0.001), and an increased INR was an independent and adjusted dose-dependent risk factor for HE among patients with liver cirrhosis.

**Conclusion:**

This finding could guide clinicians to develop individualized counseling programs and treatments for patients with HE based on the INR risk stratification.

## Introduction

Hepatic encephalopathy (HE) is an important feature of liver failure, and is defined as brain dysfunction caused by liver insufficiency and/or portal-systemic blood shunting (1). The prevalence of HE in patients with liver cirrhosis was estimated up to 20% (2, 3). Most liver cirrhotic patients need a long time to regain normal cognitive function after the onset of HE, which affected their daily life and work ability. Identifying the risk of HE can help patients adopt more stringent monitoring and lifestyle interventions, which contributed to reduce the incidence of HE.

The international normalized ratio (INR) was an indicator of severe liver injury, and was not only used as a criterion for the diagnosis of acute-on-chronic liver failure (ACLF) by the European Association for The Study of the Liver (EASL) and the Asian-Pacific Association for the Study of the Liver (APASL), but it was also a risk factor in many prognostic models of chronic liver disease, such as model for end-stage liver disease (MELD) 3.0 and chronic liver failure-consortium acute decompensation (CLIF-CAD) (4–8). However, few studies quantified the relationship between the INR and HE.

The restricted cubic spline (RCS) model can combine continuous variables and outcomes, and reflect the impact of independent variables on the risk of outcomes in the form of a continuous curve (9). It was an important method of analyzing the dose-response relationship between continuous variables and outcomes. In a cohort study on the association of the BMI with overall and cause-specific mortality, researchers found that the BMI had J-shaped associations with overall mortality and most specific causes of death and had an inverse relationship with the risk of death from mental, behavioral, and neurological diseases (10).

Although the mechanism of INR leading to HE was not clear, previous studies showed that there must be a correlation between the INR and HE (11–13). Further studies on the dose-response relationship between INR and HE would be an important step toward reducing the social burden of HE. Therefore, the present study was conducted to analyze the relationship between the INR and HE in patients with liver cirrhosis using the RCS model, and to guide clinicians to develop individualized counseling programs and treatments for patients with hepatic encephalopathy based on the INR risk stratification.

## Patients and Methods

### Data Source

We conducted a multicenter retrospective study of inpatients with liver cirrhosis at six tertiary hospitals in Chongqing, China. The study data were obtained from the electronic medical records of six tertiary hospitals on the Big Data Platform of the Medical Data Research Institute of Chongqing Medical University. The platform includes more than 40 million electronic medical records from seven tertiary hospitals in Chongqing, with the data deidentified to ensure patient privacy. Model development followed the transparent reporting of a multivariable prediction model for individual prognosis or diagnosis (TRIPOD) guidelines (14).

The study was approved by the ethics committee of Affiliated Banan Hospital of Chongqing Medical University. Since this was a retrospective study, written informed consent for participation was not required for this study in accordance with the national legislation and the institutional requirements. The need for informed consent was waived by the Ethics Committee of Affiliated Banan Hospital of Chongqing Medical University. The study was performed in accordance with relevant guidelines and regulations.

### Inclusion and Exclusion Criteria

**Figure 1:**
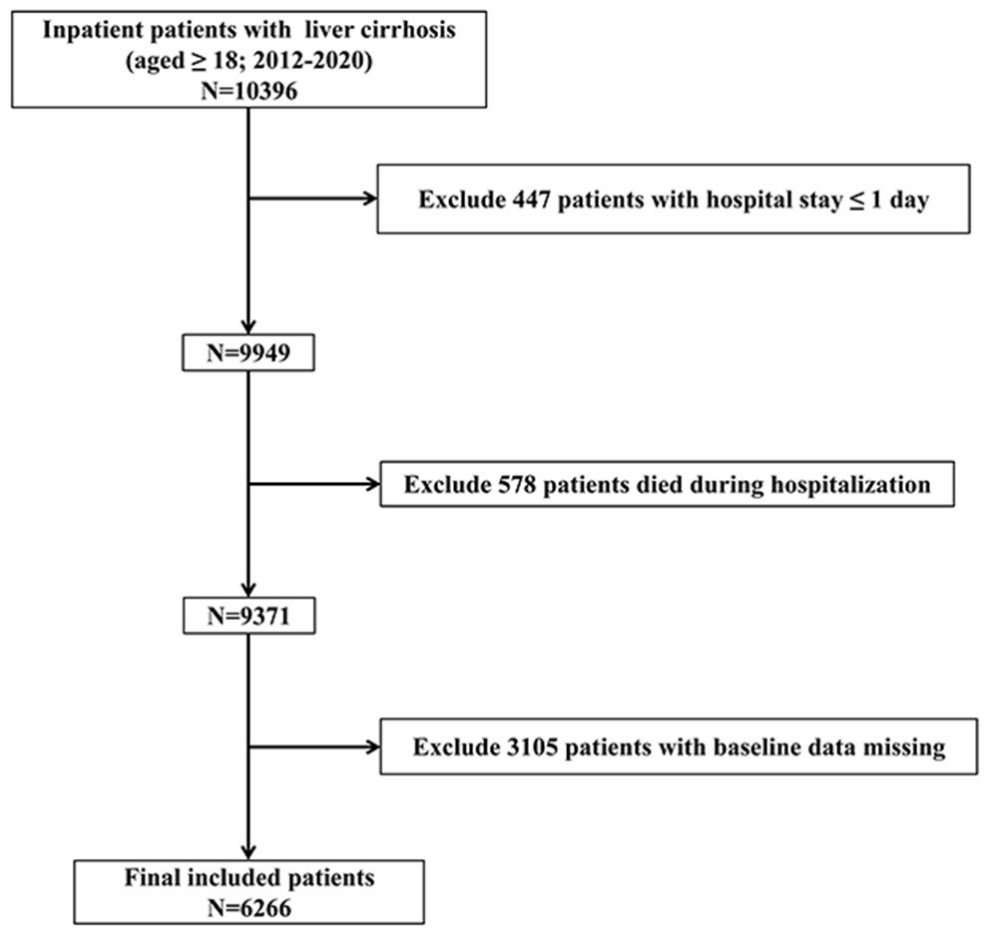
Flow of inclusions and exclusions.

The inclusion criteria were as follows: (i) data obtained from 2012 to 2020, (ii) patients aged ≥18 years, and (iii) hospitalization(s) with liver cirrhosis. The exclusion criteria were as follows: (i) hospital stay ≤ 1 day; (ii) patients died during hospitalization; and (iii) patients with baseline data missing. The study sample included 6,266 patients. The inclusion and exclusion criteria are shown in Figure 1.

### Definitions

INR was introduced as a standardized reporting mechanism allowing comparisons across laboratories and patients (15, 16). Consensus guidelines recommend that INR ≥ 1.5 can be used as a threshold, and current recommendations for targeting an INR of < 1.5 were based on studies across all surgical disciplines (17, 18). HE was a neuropsychiatric disorder that presents with a broad spectrum of cognitive and neuromuscular impairment (19). HE was classified into three types according to the etiology: type A, caused by acute liver failure; type B, caused by portosystemic shunt or shunt; and type C, caused by liver cirrhosis (20, 21).

### Data Collection

For all patients, we collected clinical data, including information related to gender, age, history of liver disease, smoking status, drinking status, alcoholic liver disease, chronic hepatitis C, chronic hepatitis B, and autoimmune liver disease as well as data related to total bilirubin (TB), neutrophil percentage (NEU%), total hemoglobin (HB), aspartate aminotransferase (AST), serum sodium (Na+), serum potassium (K+), albumin (ALB), lymphocyte percentage (LYM%), serum creatinine (SCr), red blood cell (RBC), alanine aminotransferase (ALT), and white blood cell (WBC).

### Statistical Analysis

Normally distributed continuous variables were presented as the mean ± standard deviation and analyzed using *t*-tests, whereas non-normally distributed continuous variables were presented as the median (interquartile range) and analyzed using the Mann–Whitney U rank-sum test. Categorical variables were presented as frequencies (%) and analyzed using the chi-square test or Fisher exact test. Firstly, the association between the INR and HE was investigated by using unconditional multivariable logistic regression models. Three logistic regression models were fitted. Next, RCSs were used to detect the possible non-linear dependency of the relationship between the risk of HE and INR levels, using four knots at pre-specified locations according to the percentiles of the distribution of INR, 1.0, 1.2, 1.7, and 2.6 (22). Finally, subgroup analyses were conducted to examine whether the investigated associations between INR and HE were modified by alcoholic liver disease, smoking status, and drinking status. R software (version 4.0.2, Vienna, Austria) was used to conduct the above-mentioned dose-response analyses (23). The multiple imputation method was used to fill in the missing continuous variables (24, 25). The threshold for statistical significance was set at *P* < 0.05 (two-tailed tests).

## Results

### Characteristics of the Study Population

A total of 6,266 patients were finally included in the study. The median INR of all patients was 1.4, and 70.25% of the study participants were male. The causes of cirrhosis were alcoholic liver disease (13.93%), chronic hepatitis C (4.05%), chronic hepatitis B (62.00%), and autoimmune liver disease (8.51%). The proportion of smoking (50.57%) and drinking (51.72%) in the HE group were significantly higher than those in the Non-HE group. Compared with Non-HE patients, the HE individuals had a higher INR (1.96 ± 0.69 vs. 1.49 ± 0.46, *P* < 0.001). Based on the baseline characteristics of the two cohorts of patients as listed in Table 1, significant differences were found in variables such as alcoholic liver disease, age, smoking status, drinking status, total bilirubin, neutrophil percentage, total hemoglobin, aspartate aminotransferase, serum sodium, albumin, lymphocyte percentage, serum creatinine, red blood cell, and white blood cell (*P* < 0.05).

**Table 1:** Demographic and clinical characteristics of the study population.

**Characteristics**	**Total (*n* = 6,266)**	**HE (*n* = 524)**	**Non-HE (*n* = 5,742)**	***P*-value**
Gender (*n*, %)				0.092
Male	4,402 (70.25%)	385 (73.47%)	4,017 (69.96%)	
Female	1,864 (29.95%)	139 (26.53%)	1,725 (30.04%)	
Age (IQR, years)	56.00 (49.00, 67.00)	58.00 (50.00, 67.00)	56.00 (49.00, 66.00)	0.003
International normalized ratio (*n*, %)				<0.001
≥1.5	2,418 (38.59%)	355 (67.75%)	2,063 (35.93%)	
<1.5	3,848 (61.41%)	169 (32.25%)	3,679 (64.07%)	
History of liver disease (*n*, %)				0.259
Yes	1,304 (20.81%)	99 (18.89%)	1,205 (20.99%)	
No	4,962 (79.19%)	425 (81.11%)	4,537 (79.01%)	
Smoking status (*n*, %)				0.005
Yes	2,803 (44.73%)	265 (50.57%)	2,538 (44.20%)	
No	3,463 (55.27%)	259 (49.43%)	3,204 (55.80%)	
Drinking status (*n*, %)				0.001
Yes	2,792 (44.56%)	271 (51.72%)	2,521 (43.90%)	
No	3,474 (55.44%)	253 (48.28%)	3,221 (56.10%)	
Alcoholic liver disease (*n*, %)				<0.001
Yes	873 (13.93%)	129 (24.62%)	744 (12.96%)	
No	5,393 (86.07%)	395 (75.38%)	4,998 (87.04%)	
Chronic hepatitis C (*n*, %)				0.684
Yes	254 (4.05%)	23 (4.39%)	231 (4.02%)	
No	6,012 (95.95%)	501 (95.61%)	5,511 (95.98%)	
Chronic hepatitis B (*n*, %)				0.226
Yes	3,885 (62.00%)	312 (59.54%)	3,573 (62.23%)	
No	2,381 (38.00%)	212 (40.46%)	2,169 (37.77%)	
Autoimmune liver disease (*n*, %)				0.293
Yes	533 (8.51%)	51 (9.73%)	482 (8.39%)	
No	5,733 (91.49%)	473 (90.27%)	5,260 (91.61%)	
TB (IQR, umol/l)	31.70 (17.80, 84.33)	68.40 (31.78, 185.00)	30.00 (17.20, 76.58)	<0.001
NEU% (IQR, %)	68.44 (59.40, 76.93)	73.70 (63.80, 81.46)	68.00 (59.00, 76.51)	<0.001
HB (IQR, g/L)	108.00 (85.00, 127.00)	101.00 (79.00, 120.00)	109.00 (85.00, 128.00)	<0.001
AST (IQR, IU/L)	59.00 (34.00, 119.00)	66.35 (41.00, 142.25)	58.00 (33.00, 117.00)	<0.001
Na+ (IQR, mmol/l)	139.10 (136.20, 141.60)	138.00 (133.98, 141.13)	139.20 (136.50, 141.60)	<0.001
K+ (IQR, mmol/l)	3.85 (3.53, 4.17)	3.87 (3.48, 4.32)	3.85 (3.54, 4.16)	0.223
ALB (IQR, g/L)	30.80 (27.00, 35.20)	28.70 (25.68, 31.60)	31.10 (27.10, 35.50)	<0.001
LYM% (IQR, %)	20.70 (13.82, 28.40)	16.37 (10.02, 22.71)	21.10 (14.30, 28.80)	<0.001
SCr (IQR, umol/l)	66.10 (55.40, 81.00)	67.70 (56.80, 94.08)	66.00 (55.30, 80.10)	<0.001
RBC (IQR, × 10^12^/L)	3.50 (2.87, 4.10)	3.23 (2.71, 3.80)	3.53 (2.89, 4.11)	<0.001
ALT (IQR, IU/L)	38.00 (22.00, 89.00)	38.00 (23.40, 87.25)	38.00 (22.00, 89.00)	0.272
WBC (IQR, × 10^9^/L)	4.42 (3.06, 6.43)	5.04 (3.38, 8.09)	4.35 (3.01, 6.30)	<0.001

*TB, total bilirubin; NEU%, Neutrophil percentage; HB, Total hemoglobin; AST, aspartate aminotransferase; Na+, Serum sodium; K+, Serum potassium; ALB, Albumin; LYM%, Lymphocyte percentage; SCr, serum creatinine; RBC, Red blood cell; ALT, Alanine aminotransferase; WBC, white blood cell; IQR, interquartile range*.

### Univariate and Multivariate Logistic Regression Analyses for an Investigation of the Association Between the INR and HE

As shown in Table 2, the INR levels were significantly associated with HE in total subjects and in the subgroups stratified by alcoholic liver disease (yes/no), smoking status (yes/no) and drinking status (yes/no) *via* unadjusted logistic regression and multivariate logistic regression (*P* < 0.001). After adjusting for alcoholic liver disease, age, smoking status, drinking status, total bilirubin, neutrophil percentage, total hemoglobin, aspartate aminotransferase, serum sodium, albumin, lymphocyte percentage, serum creatinine, red blood cell, and white blood cell, INR ≥ 1.5 (total: OR = 2.606, 95% CI: 2.072–3.278, *P* < 0.001; non-alcoholic liver disease: OR = 2.667, 95% CI: 2.043–3.482, *P* < 0.001; alcoholic liver disease: OR = 2.380, 95% CI: 1.504–3.765, *P* < 0.001; non-smoker: OR = 2.385, 95% CI: 1.729–3.289, *P* < 0.001; smoker: OR = 2.826, 95% CI: 2.037–3.921, *P* < 0.001; non-drinker: OR = 2.512, 95% CI: 1.811–3.484, *P* < 0.001; drinker: OR = 2.691, 95% CI: 1.951–3.712, *P* < 0.001) was independently associated with increased risk of HE.

**Table 2:** Logistic regression analysis of the association between the INR and HE.

	**Model** ^**a**^		**Model** ^**b**^		**Model** ^**c**^	
	**OR (95% CI)**	***P-*value**	**OR (95% CI)**	***P*-value**	**OR (95% CI)**	***P*-value**
**Total**						
INR < 1.5	Ref (1.000)	/	Ref (1.000)	/	Ref (1.000)	/
INR ≥ 1.5	3.747 (3.098, 4.532)	<0.001	3.995 (3.290, 4.850)	<0.001	2.606 (2.072, 3.278)	<0.001
**Alcoholic liver disease**						
**Yes**						
INR < 1.5	Ref (1.000)	/	Ref (1.000)	/	Ref (1.000)	/
INR ≥ 1.5	3.059 (2.071, 4.518)	<0.001	3.209 (2.160, 4.768)	<0.001	2.380 (1.504, 3.765)	<0.001
**Alcoholic liver disease**						
**No**						
INR < 1.5	Ref (1.000)	/	Ref (1.000)	/	Ref (1.000)	/
INR ≥ 1.5	3.975 (3.185, 4.960)	<0.001	4.272 (3.416, 5.341)	<0.001	2.667 (2.043, 3.482)	<0.001
**Smoking status**						
**Yes**						
INR < 1.5	Ref (1.000)	/	Ref (1.000)	/	Ref (1.000)	/
INR 1.5	3.877 (2.946, 5.101)	<0.001	4.125 (3.117, 5.459)	<0.001	2.826 (2.037, 3.921)	<0.001
**Smoking status**						
**No**						
INR < 1.5	Ref (1.000)	/	Ref (1.000)	/	Ref (1.000)	/
INR ≥ 1.5	3.572 (2.736, 4.663)	<0.001	3.881 (2.961, 5.086)	<0.001	2.385 (1.729, 3.289)	<0.001
**Drinking status**						
**Yes**						
INR < 1.5	Ref (1.000)	/	Ref (1.000)	/	Ref (1.000)	
INR ≥ 1.5	3.607 (2.753, 4.728)	<0.001	3.865 (2.932, 5.096)	<0.001	2.691 (1.951, 3.712)	<0.001
**Drinking status**						
**No**						
INR < 1.5	Ref (1.000)	/	Ref (1.000)	/	Ref (1.000)	/
INR ≥ 1.5	3.781 (2.885, 4.955)	<0.001	4.125 (3.135, 5.427)	<0.001	2.512 (1.811, 3.484)	<0.001

*Model^a^, unadjuested. Model^b^, adjusted for alcoholic liver disease, smoking status, drinking status, age. Model^c^, adjusted for alcoholic liver disease, smoking status, drinking status, age, total bilirubin, neutrophil percentage, total hemoglobin, aspartate aminotransferase, serum sodium, albumin, lymphocyte percentage, serum creatinine, red blood cell, and white blood cell*.

### RCS Analysis for the Dose-Response Relationship Between the INR and HE

**Figure 2:**
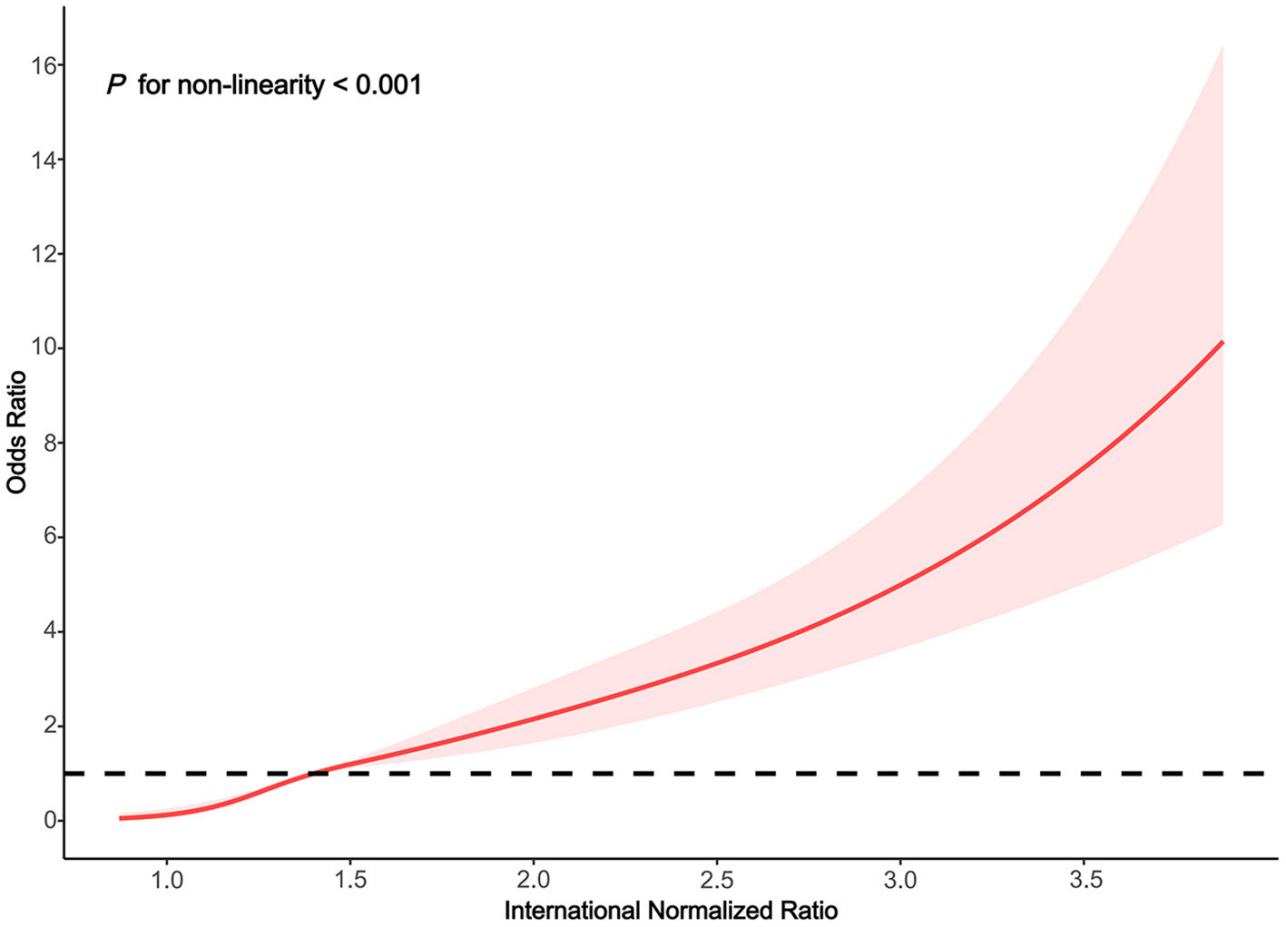
Association between the INR and HE risk based on restricted cubic spline model in total population.

We used the RCS model with four knots to simulate the relationship between the INR and the risk for HE. After adjusting for alcoholic liver disease, age, smoking status, drinking status, total bilirubin, neutrophil percentage, total hemoglobin, aspartate aminotransferase, serum sodium, albumin, lymphocyte percentage, creatinine, red blood cell, and white blood cell, the RCS model showed a non-linear relationship between INR classification and HE (Figure 2 non-linear test, χ^2^ = 30.940, *P*_non−linearity_ < 0.001). With an INR of 1.4 as a reference, the ORs (95% CI) of the four knots of INR were 0.13 (0.07–0.27) for 1.0, 0.47 (0.38–0.58) for 1.2, 1.56 (1.30–1.88) for 1.7, and 3.64 (2.75–4.83) for 2.6.

### Subgroup Analyses

We performed subgroups analyses to stratify the association between the INR and HE by alcoholic liver disease, smoking status and drinking status.

**Figure 3:**
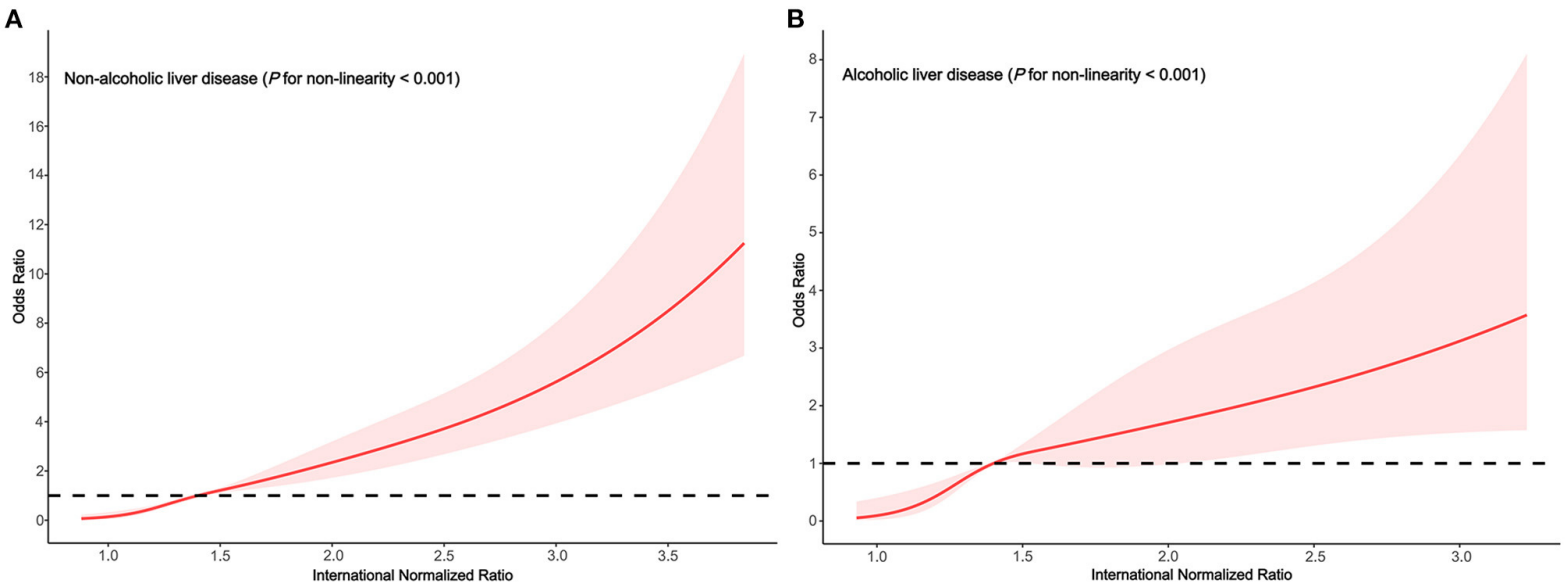
Association between the INR and HE risk based on restricted cubic spline model stratified by alcoholic liver disease **(A)** no; **(B)** yes.

In the subgroups stratified by alcoholic liver disease, the fitted dose-response relationships were described in Figure 2. In the group non-alcoholic liver disease (Figure 3A *P*_non−linearity_ < 0.001) with an INR of 1.4 as a reference, the ORs (95% CI) of the four knots of INR were 0.15 (0.07–0.34) for 1.0, 0.50 (0.40–0.62) for 1.2, 1.67 (1.34–2.07) for 1.7, and 4.05 (2.92–5.62) for 2.6. In the group alcoholic liver disease (Figure 3B *P*_non−linearity_ = 0.008) with an INR of 1.4 as a reference, the ORs (95% CI) of the four knots of INR were 0.10 (0.03–0.41) for 1.0, 0.44 (0.29–0.68) for 1.2, 1.38 (0.93–2.05) for 1.7, and 2.47 (1.37–4.46) for 2.6.

**Figure 4:**
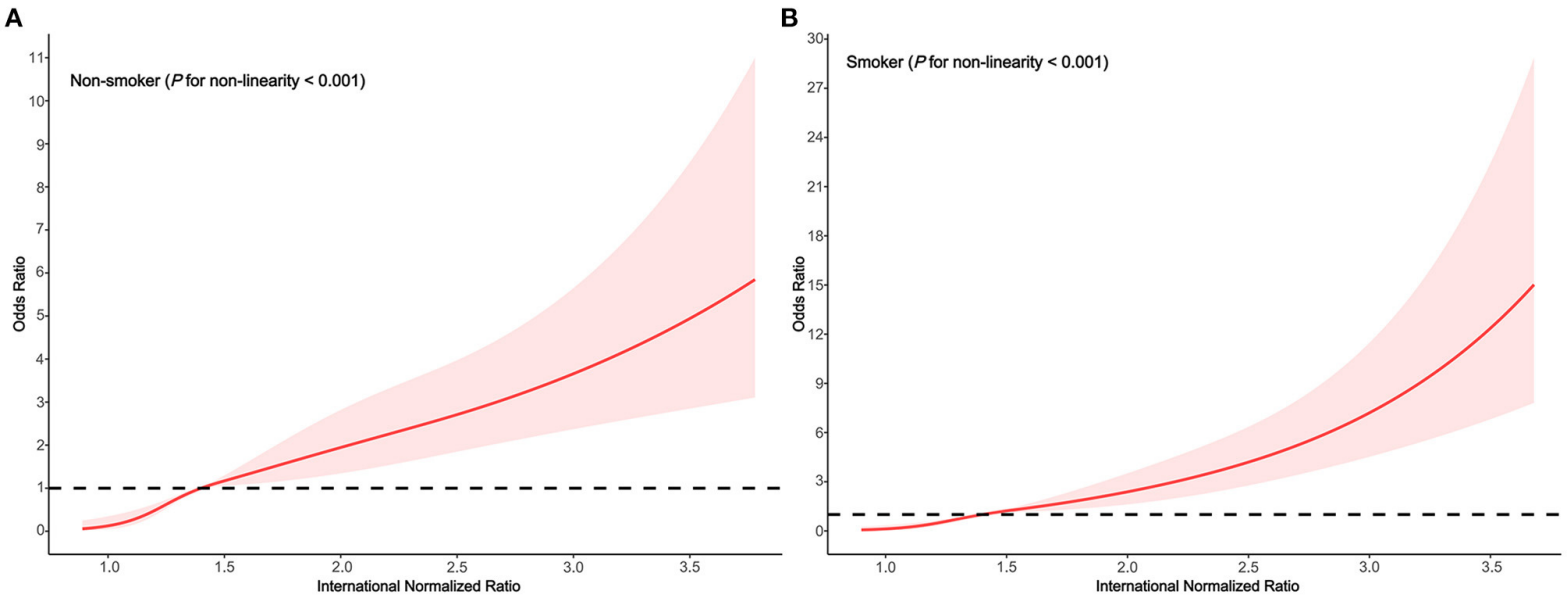
Association between the INR and HE risk based on restricted cubic spline model stratified by smoking status **(A)** no; **(B)** yes.

In the subgroups stratified by smoking status, the fitted dose-response relationships were described in Figure 3. In the group non-smoker (Figure 4A *P*_non−linearity_ < 0.001) with an INR of 1.4 as a reference, the ORs (95% CI) of the four knots of INR were 0.13 (0.05–0.35) for 1.0, 0.51 (0.40–0.65) for 1.2, 1.49 (1.14–1.94) for 1.7, and 2.88(1.96–4.24) for 2.6. In the group smoker (Figure 4B *P*_non−linearity_ = 0.002) with an INR of 1.4 as a reference, the ORs (95% CI) of the four knots of INR were 0.13 (0.05–0.35) for 1.0, 0.46 (0.34–0.63) for 1.2, 1.65 (1.26–2.16) for 1.7, and 4.70 (3.10–7.13) for 2.6.

**Figure 5:**
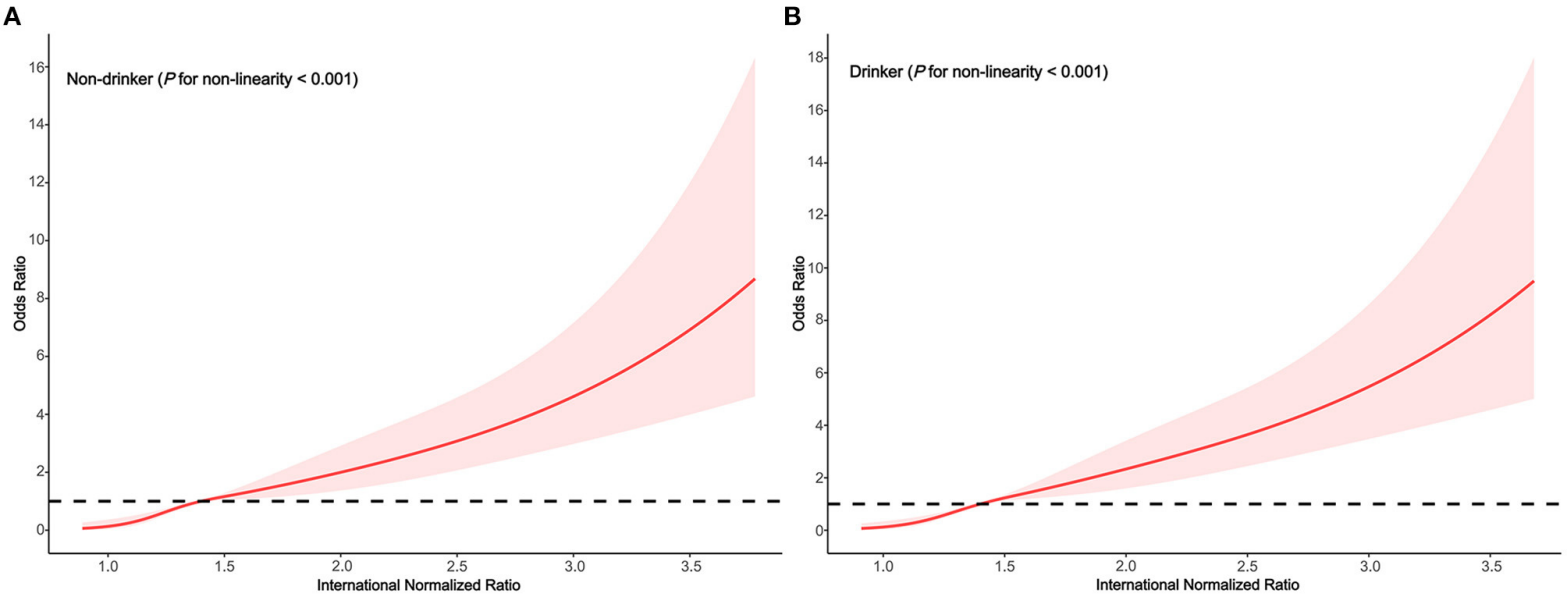
Association between the INR and HE risk based on restricted cubic spline model stratified by drinking status **(A)** no; **(B)** yes.

In the subgroups stratified by drinking status, the fitted dose-response relationships were described in Figure 4. In the group non-drinker (Figure 5A *P*_non−linearity_ = 0.001) with an INR of 1.4 as a reference, the ORs (95% CI) of the four knots of INR were 0.14 (0.05–0.37) for 1.0, 0.52 (0.41–0.67) for 1.2, 1.48 (1.13–1.92) for 1.7, and 3.35 (2.25–4.99) for 2.6. In the group drinker (Figure 5B *P*_non−linearity_ < 0.001) with an INR of 1.4 as a reference, the ORs (95% CI) of the four knots of INR were 0.13 (0.05–0.35) for 1.0, 0.44 (0.32–0.61) for 1.2, 1.66 (1.28–2.15) for 1.7, and 3.98 (2.66–6.00) for 2.6.

## Discussion

HE is a common complication of cirrhosis that is associated with a poor prognosis. Presently, there is still a lack of quantitative indicators for the risk of HE (26). In this study, the clinical data of 6,266 hospitalized patients with liver cirrhosis were analyzed, alcoholic liver disease, age, smoking status, drinking status, total bilirubin, neutrophil percentage, total hemoglobin, aspartate aminotransferase, serum sodium, albumin, lymphocyte percentage, serum creatinine, red blood cell, and white blood cell were all associated with HE, which was consistent with the results of previous studies (27–29). We also found that in patients with liver cirrhosis, INR ≥ 1.5 was significantly more relevant for HE incidence than INR < 1.5. After adjustment for confounding factors, the dose-response analysis suggested that higher INR level was an independent, dose-dependent risk factor for HE (non-linear test, χ^2^ = 30.940, *P*_non−linearity_ < 0.001), and strategies to prevent HE with a focus on continuous changes in INR should be emphasized.

In a study aimed at investigating the quantitative relationship between INR and short-term prognosis in hospitalized patients with cirrhosis or advanced fibrosis, the researchers found that in patients with advanced fibrosis, the peak value of the second derivative of 90-day liver transplant-free mortality was at an INR level of 1.7, and the valley value was achieved when INR was 2.7, indicating that the fastest change in mortality occured when INR was between 1.7 and 2.7 (30). In our study, there was a similar relationship between INR and HE. With an INR of 1.4 as a reference, the HE ORs (95% CI) were 1.56 (1.30–1.88) and 3.64 (2.75–4.83) at INR level of 1.7 and 2.6, respectively. In patients with cirrhosis or non-cirrhotic chronic liver disease, INR ≥ 1.5 was considered important for the diagnosis of ACLF by the APASL (6). In the EASL, INR ≥ 2.5 was an important indicator of ACLF (4). The INR reference value obtained in this study was 1.4, which was slightly lower than the classical threshold of 1.5, possibly due to the different study populations. With the increase of INR, the dose-response relationship between the INR and HE showed a non-linear increase, and INR > 1.4 may be used to identify patients with early potential HE. Further research is needed to confirm this result in the future.

From the pathophysiological point of view, it was well-known that an increase in the INR indirectly reflected insufficient liver function reserve in patients with liver cirrhosis and was also a predictor of variceal bleeding (31). A recent study had shown that acute variceal bleeding (AVB) was the most important factor associated with risk of overt HE. After the recurrence of AVB, decompensated cirrhosis (Child B or C) was the second most important factor contributed to the development of overt HE (32). Shalimar and Acharya mentioned that coagulation dysfunction should be actively corrected in the management of HE to achieve the goal of INR < 1.7 (33). A study revealed that the incidence of early readmission was significantly higher in patients with HE with an INR > 1.62 at discharge than in those with an INR ≤ 1.62 (44% vs 19%; *P* < 0.001) (34). In addition, the INR and HE were included in the APASL ACLF Research Consortium (AARC) score used to manage APASL-ACLF, confirming again the prognostic importance of the above indicators (35).

The advantages of this study included the availability of clinical electronic medical record data, which integrated confounding factors such as social and demographic characteristics, etiology and laboratory indicators, and combined treatment data from six medical institutions, partially overcoming the bias caused by single-center data. From a statistical methodological point of view, the relationship between INR and HE was well-fitted by combining the unconditional multivariate logistic regression model with the RCS model, instead of subjectively classifying the INR into different ranges, which objectively depicts the non-linear dose-response relationship between continuous changes in the INR and HE.

There were also some limitations in this study. First, there might be selection bias due to the retrospective study design. However, our research tried to minimize bias based on retrieving records from a comprehensive multicenter database. Second, the data on education level, economic level, marital status, and occupation were not available in this study. Further research is warranted to explore the impact of these important indicators.

## Conclusion

A dose-response relationship exists between the INR and HE, and an increased INR was an independent and adjusted dose-dependent risk factor for HE among patients with liver cirrhosis. This finding can help clinicians to predict the risk of hepatic encephalopathy from objective indicators, and the quantitative indicators are more helpful for patients to understand the disease and cooperate with treatment, so as to identify and treat the disease at an early stage and avoid the occurrence of serious events.

## Data Availability Statement

The raw data supporting the conclusions of this article will be made available by the authors, without undue reservation.

## Author Contributions

JT and XX designed the research. JT, YY, QZ, and QX collected and organized data. JT, ZL, and YH analyzed the data. JT drafted the manuscript. XX contributed to the critical revision of the manuscript. All authors contributed to the manuscript and approved the submitted version.

## Funding

This study was funded by Hospital Infection Prevention and Control Project (Grant No. SCGK202115).

## Conflict of Interest

The authors declare that the research was conducted in the absence of any commercial or financial relationships that could be construed as a potential conflict of interest.

## Publisher's Note

All claims expressed in this article are solely those of the authors and do not necessarily represent those of their affiliated organizations, or those of the publisher, the editors and the reviewers. Any product that may be evaluated in this article, or claim that may be made by its manufacturer, is not guaranteed or endorsed by the publisher.

## References

[1] Karin W. Hepatic encephalopathy: definition, clinical grading and diagnostic principles. Drugs. (2019) 79:5–9. 10.1007/s40265-018-1018-z 30706420 PMC6416238

[2] Anuj B Thomas W Samuel H Ryma T Ann F Marcus R. Prognostic significance of hepatic encephalopathy in patients with cirrhosis treated with current standards of care. World J Gastroenterol. (2020) 26:2221–31. 10.3748/wjg.v26.i18.2221 32476788 PMC7235207

[3] Labenz C Toenges G Huber Y Nagel M Marquardt JU Schattenberg JM. Raised serum interleukin-6 identifies patients with liver cirrhosis at high risk for overt hepatic encephalopathy. Aliment Pharmacol Ther. (2019) 50:1112–9. 10.1111/apt.15515 31583743

[4] Moreau R Jalan R Gines P Pavesi M Angeli P Cordoba J. Acute-on-Chronic liver failure is a distinct syndrome that develops in patients with acute decompensation of cirrhosis. Gastroenterology. (2013) 144:1426–37. 10.1053/j.gastro.2013.02.042 23474284

[5] Mahmud N Kaplan DE Taddei TH Goldberg DS. Incidence and mortality of acute-on-chronic liver failure using two definitions in patients with compensated cirrhosis. Hepatology. (2019) 69:2150–63. 10.1002/hep.30494 30615211 PMC6461492

[6] Sarin SK Choudhury A Sharma MK Maiwall R Al Mahtab M Rahman S. Acute-on-chronic liver failure: consensus recommendations of the Asian Pacific association for the study of the liver (APASL): an update. Hepatol Int. (2019) 13:353–90. 10.1007/s12072-019-09946-3 31172417 PMC6728300

[7] Kim WR Mannalithara A Heimbach JK Kamath PS Asrani SK Biggins SW. MELD 3.0: the model for end-stage liver disease updated for the modern era. Gastroenterology. (2021) 161:1887–95. 10.1053/j.gastro.2021.08.050 34481845 PMC8608337

[8] Jalan R Pavesi M Saliba F Amorós A Fernandez J Holland-Fischer P. The CLIF consortium acute decompensation score (CLIF-C ADs) for prognosis of hospitalised cirrhotic patients without acute-on-chronic liver failure. J Hepatol. (2015) 62:831–40. 10.1016/j.jhep.2014.11.012 25463539

[9] van Dijk SC Sohl E Oudshoorn C Enneman AW Ham AC Swart KM. Non-linear associations between serum 25-OH vitamin D and indices of arterial stiffness and arteriosclerosis in an older population. Age Ageing. (2015) 44:136–42. 10.1093/ageing/afu095 25038832

[10] Bhaskaran K dos-Santos-Silva I Leon DA Douglas IJ Smeeth L. Association of BMI with overall and cause-specific mortality: a population-based cohort study of 3·6 million adults in the UK. Lancet Diabetes Endocrinol. (2018) 6:944–53. 10.1016/S2213-8587(18)30288-2 30389323 PMC6249991

[11] Amoako D Adwoa A Foster O Francisca D Peprah AB. Sociodemographic characteristics, complications requiring hospital admission and causes of in-hospital death in patients with liver cirrhosis admitted at a district hospital in Ghana. PLoS ONE. (2021) 16:e0253759. 10.1371/journal.pone.0253759 34166471 PMC8224881

[12] Mahmud N Forde KA. Autoimmunity in indeterminate etiologies of acute liver failure: is this autoimmune liver disease or an autoimmune phenotype to drug toxicity? Liver Transpl. (2020) 26:743–5. 10.1002/lt.25776 32279421

[13] Gao F Zhang Q Liu Y Gong G Mao D Gong Z. Nomogram prediction of individual prognosis of patients with acute-on-chronic hepatitis B liver failure. Digest Liver Dis. (2018) 51:425–33. 10.1016/j.dld.2018.08.023 30241795

[14] Collins GS Reitsma JB Altman DG Moons KG. Transparent reporting of a multivariable prediction model for individual prognosis or diagnosis (TRIPOD): the TRIPOD statement. BMJ. (2015) 350:g7594. 10.1136/bmj.g7594 25569120

[15] Dorgalaleh A Favaloro EJ Bahraini M Rad F. Standardization of prothrombin time/international normalized ratio (PT/INR). Int J Lab Hematol. (2020) 43:21–8. 10.1111/ijlh.13349 32979036

[16] Jacobson AK. Warfarin monitoring: point-of-care INR testing limitations and interpretation of the prothrombin time. J Thromb Thrombolysis. (2008) 25:10–1. 10.1007/s11239-007-0098-5 18008039

[17] Wendon J Cordoba J Dhawan A Larsen FS Manns M Nevens F. EASL clinical practical guidelines on the management of acute (fulminant) liver failure. J Hepatol. (2017) 66:1047–81. 10.1016/j.jhep.2016.12.003 28417882

[18] Rudasill SE Liu J Kamath AF. Revisiting the international normalized ratio (INR) threshold for complications in primary total knee arthroplasty: an analysis of 21,239 cases. J Bone Joint Surg Am. (2019) 101:514–22. 10.2106/JBJS.18.00771 30893232

[19] Robin G Alexander Z Macarena ST Franz S Christiane L Andreas W. Covert hepatic encephalopathy and spontaneous portosystemic shunts increase the risk of developing overt hepatic encephalopathy. Liver Int. (2020) 40:3093–102. 10.1111/liv.14660 32890428

[20] Vilstrup H Amodio P Bajaj J Cordoba J Ferenci P Mullen KD. Hepatic encephalopathy in chronic liver disease: 2014 practice guideline by the American association for the study of liver diseases and the European association for the study of the liver. Hepatology. (2014) 60:715–35. 10.1002/hep.27210 25042402

[21] Davis BC Bajaj JS. Effects of alcohol on the brain in cirrhosis: beyond hepatic encephalopathy. Alcohol Clin Exp Res. (2018) 42:660–7. 10.1111/acer.13605 29417604

[22] Xueshan J Tingting D Yufen H Zhongyi Y Pingyang Z Jingchao R. Identifying the dose response relationship between seminal metal at low levels and semen quality using restricted cubic spline function. Chemosphere. (2022) 295:133805. 10.1016/j.chemosphere.2022.133805 35134404

[23] Yixian X Didi H Fengshuo X Si S Xinkai Z Hao W. Using restricted cubic splines to study the duration of antibiotic use in the prognosis of ventilator-associated pneumonia. Front Pharmacol. (2022) 13:898630. 10.3389/fphar.2022.898630 35571078 PMC9099062

[24] Templ M Alfons A Filzmoser P. Exploring incomplete data using visualization techniques. Adv Data Anal Classif. (2012) 6:29–47. 10.1007/s11634-011-0102-y

[25] Zhongheng Z. Multiple imputation with multivariate imputation by chained equation (MICE) package. Ann Transl Med. (2016) 4:30. 10.3978/j.issn.2305-5839.2015.12.63 26889483 PMC4731595

[26] Shawcross DL Dunk AA Jalan R Kircheis G De Knegt RJ Laleman W. How to diagnose and manage hepatic encephalopathy: a consensus statement on roles and responsibilities beyond the liver specialist. Eur J Gastroenterol Hepatol. (2016) 28:146–52. 10.1097/MEG.0000000000000529 26600154 PMC4885589

[27] Limin T Meng Z Xiulian L Lijuan Z. Glucuronidated bilirubin: significantly increased in hepatic encephalopathy. Prog Mol Biol Transl Sci. (2019) 162:363–76. 10.1016/bs.pmbts.2018.12.009 30905463

[28] Lars B Pere G Hendrik V Hugh W Peter J. Serum sodium as a risk factor for hepatic encephalopathy in patients with cirrhosis and ascites. J Gastroenterol Hepatol. (2019) 34:914–20. 10.1111/jgh.14558 30500090

[29] Bai Z Guo X Tacke F Li Y Li H Qi X. Association of serum albumin level with incidence and mortality of overt hepatic encephalopathy in cirrhosis during hospitalization. Ther Adv Gastroenterol. (2019) 12:1756284819881302. 10.1177/1756284819881302 31636711 PMC6783662

[30] Ying W Fuchen D Shuning S Xianbo W Xin Z Yan H. Increased INR values predict accelerating deterioration and high short-term mortality among patients hospitalized with cirrhosis or advanced fibrosis. Front Med. (2021) 8:762291. 10.3389/fmed.2021.762291 34869468 PMC8637055

[31] Daniela M Ioana G Bogdan F Lidia P Cristina L Andrei C. Predictors of variceal or nonvariceal source of upper gastrointestinal bleeding. An etiology predictive score established and validated in a tertiary referral center. J Gastrointest Liver Dis. (2013) 22:379–84. 10.1007/s00535-013-0761-x 24369318

[32] Higuera-de-la-Tijera F Servín-Caamaño AI Salas-Gordillo F Pérez-Hernández JL Abdo-Francis JM Camacho-Aguilera J. Primary prophylaxis to prevent the development of hepatic encephalopathy in cirrhotic patients with acute variceal bleeding. Can J Gastroenterol Hepatol. (2018) 2018:3015891. 10.1155/2018/3015891 30079329 PMC6069577

[33] Shalimar Acharya SK. Management in acute liver failure. J Clin Exp Hepatol. (2015) 5 (Suppl. 1):104–15. 10.1016/j.jceh.2014.11.005 26041950 PMC4442864

[34] Xiao-Peng H Jian G. International normalized ratio and model for end-stage liver disease score predict short-term outcome in cirrhotic patients after the resolution of hepatic encephalopathy. World J Gastroenterol. (2019) 25:3426–37. 10.3748/wjg.v25.i26.3426 31341366 PMC6639559

[35] Choudhury A Jindal A Maiwall R Sharma MK Sharma BC Pamecha V. Liver failure determines the outcome in patients of acute-on-chronic liver failure (ACLF): comparison of APASL ACLF research consortium (AARC) and CLIF-SOFA models. Hepatol Int. (2017) 11:461–71. 10.1007/s12072-017-9816-z 28856540

